# Synergistic mortality risk of glycemic and blood pressure variability in critical stroke: A retrospective cohort study from the MIMIC-IV database

**DOI:** 10.1097/MD.0000000000049291

**Published:** 2026-06-26

**Authors:** Chanchan Miao, Lele Kang, Xuejun Gao

**Affiliations:** a Department of Neurology, Yan‘an University Affiliated Hospital, Yan’an, Shaanxi, China.

**Keywords:** glycemic variability, MIMIC-IV database, mortality, stroke, systolic blood pressure variability

## Abstract

Variability in glycemic (GV) and systolic blood pressure (SBPV) are independent risk factors for adverse outcomes in stroke. However, their combined impact on mortality in stroke patients requiring intensive care remains unclear. This study aimed to evaluate the individual and additive effects of short-term GV and SBPV on mortality in critically ill stroke patients. We retrospectively analyzed hemorrhagic and ischemic stroke patients from the Medical Information Mart for Intensive Care IV database. GV and SBPV were calculated as the coefficient of variation from all glucose and systolic blood pressure measurements within the first 72 hours of intensive care unit admission. High variability was defined as the highest tertile. The additive effect was assessed by the number of high-variability parameters and a composite variability score (0–4). The study included 2659 hemorrhagic and 3606 ischemic stroke patients. After full adjustment, the highest GV tertile was associated with significantly increased 28-day mortality (Hemorrhagic: hazard ratio [HR] = 1.84, 95% confidence interval [CI] 1.38–2.44; Ischemic: HR = 1.35, 95% CI 1.08–1.70). Similarly, the highest SBPV tertile was associated with higher mortality (Ischemic: HR = 1.67, 95% CI 1.35–2.06). Most importantly, patients with both high GV and high SBPV had the most significant risk, with adjusted HRs of 2.51 (95% CI 1.89–3.33) for 28-day mortality in hemorrhagic stroke and 2.01 (95% CI 1.57–2.58) in ischemic stroke. A graded, dose–response relationship was observed between increasing variability score and mortality risk (*P* for trend < .001). Short-term GV and SBPV during intensive care unit stay are independent and additive predictors of mortality in critically ill stroke patients. Stabilizing these parameters may be a potential therapeutic target to improve outcomes.

## 1. Introduction

Stroke represents a formidable global health challenge, standing as a leading cause of mortality and permanent disability worldwide.^[1]^ The condition is marked by high incidence, severe disability, and substantial economic costs, imposing a heavy burden on patients and society.^[2]^ In China, stroke burden remains particularly heavy: in 2020, there were approximately 3.4 million new strokes and 1.6 million stroke-related deaths, making stroke the leading cause of death and adult disability nationwide.^[3]^ This highlights the crucial need for refined risk stratification tools and a more comprehensive understanding of prognostic determinants in cerebrovascular disease.

While the management of traditional risk factors remains paramount, emerging evidence highlights that the variability of key physiological parameters may offer profound insights beyond their absolute levels. In particular, glycemic variability (GV) and systolic blood pressure variability (SBPV) have garnered significant attention as dynamic risk markers.^[4–7]^ Substantial research now confirms that GV and SBPV are not mere epiphenomena but function as independent cardiovascular risk factors, with predictive values distinct from those of average glucose and systolic blood pressure (SBP) levels.^[8–10]^ Consequently, a growing body of research has focused to elucidating how these fluctuations influence stroke incidence and outcomes.

Notably, foundational work in ambulatory and chronically ill populations has demonstrated that long-term, visit-to-visit GV and SBPV can additively increase the risk of major adverse cardiovascular events, including stroke.^[11–13]^ This compelling evidence for synergistic harm, however, originates from a context fundamentally different from the acute, high-stakes environment of the intensive care unit (ICU). The injured brain following a stroke is exquisitely vulnerable to secondary insults, rendering physiological stability during the acute phase potentially critical. However, it remains unclear whether short-term GV and SBPV, measured during the initial ICU stay, exert a similar, additive detrimental effect on mortality. Furthermore, the pathophysiological mechanisms through which these acute metabolic and hemodynamic instabilities interact to influence cerebrovascular outcomes remain poorly elucidated.

To address these knowledge gaps, this retrospective cohort study utilized the Medical Information Mart for Intensive Care IV (MIMIC-IV) database to analyze critically ill patients with stroke. The study had 2 primary objectives: first, to rigorously evaluate the individual and joint effects of short-term GV and SBPV on 28-day and 1-year mortality; second, to develop a novel composite variability score (VS) that quantifies the cumulative prognostic impact of these 2 physiological instabilities in the critically ill stroke population.

## 2. Methods

### 2.1. Data source

This retrospective study used version 2.2 of the MIMIC-IV database,^[14]^ a public repository containing over 50,000 de-identified patient records from Beth Israel Deaconess Medical Center collected between 2008 and 2019.^[14,15]^ The Institutional Review Boards of the Massachusetts Institute of Technology and Beth Israel Deaconess Medical Center approved the creation of the database. One author (C.C. Miao) accessed MIMIC-IV under certification number 55642259. The study adhered to the Declaration of Helsinki. Individual informed consent was waived because all data are de-identified.

### 2.2. Study population

This retrospective study included patients diagnosed with nontraumatic cerebral hemorrhage or cerebral infarction according to the International Classification of Diseases (ICD-9 or ICD-10). Exclusion criteria for hemorrhagic stroke: age < 18 years (n = 0); ICU stay < 24 hours (n = 178); < 3 glucose measurements within 72 hours (n = 987); and SBP measured < 10 times within 72 hours (n = 714). Exclusion criteria for ischemic stroke: age < 18 years (n = 0); ICU stay < 24 hours (n = 331); glucose measurement < 3 times within 72 hours (n = 1021); and measurement of SBP < 10 times within 72 hours (n = 659). Only information from the first admission was collected for patients with repeated hospitalizations. Ultimately, data were obtained from the database for 2659 hemorrhagic stroke patients and 3606 ischemic stroke patients. The specific inclusion and exclusion process is detailed in Supplementary Figure 1, Supplemental Digital Content 1.

### 2.3. Data extraction and definitions

We extracted patient demographics, comorbidities, laboratory tests, and treatment data from the MIMIC-IV database using the MIT Laboratory for Computational Physiology (https://github.com/MIT-LCP/mimic-code). This study included only patients with more than 3 glucose measurements and more than 10 SBP readings to reduce bias from limited data.^[4,6,7]^ We also extracted all glucose and SBP values needed to calculate glucose variability (GV), SBPV, mean glucose, and mean SBP. Data were retrieved using Navicat Premium (v15.0.29) and SQL.

Because there is no gold standard for measuring GV and SBPV, we used the coefficient of variation (CV = standard deviation/mean × 100%) for all readings during the first 72 hours of ICU stay.^[6,7]^ We excluded SBP values above 300 mm Hg or below 40 mm Hg as outliers.^[5,6]^ Glucose sampling frequency and timing were guided by clinical judgment. Antihypertensive drugs included ACE inhibitors, angiotensin receptor blockers, α-blockers, β-blockers, calcium-channel blockers, and diuretics. Antidiabetic treatments comprised insulin, oral agents, GLP-1 receptor agonists, and SGLT-2 inhibitors (e.g., metformin, sulfonylureas, thiazolidinediones, DPP-4 inhibitors, and α-glucosidase inhibitors). Antiplatelet agents included enteric-coated aspirin, clopidogrel, ticagrelor, and indobufen. Hypoglycemia was defined as glucose < 70 mg/dL.

### 2.4. Study endpoints

The primary endpoint was 28-day in-hospital mortality, and the secondary endpoint was 1-year mortality. In-hospital deaths were identified using the hospital_expire_flag in the admissions table. Dates of death were obtained from the patients table and linked to hospital and state records. Complete 1-year follow-up data were available for all patients.

### 2.5. Statistical analysis

For continuous variables, data are presented as mean ± standard deviation for normally distributed variables or median (interquartile range) for non-normally distributed variables. Categorical variables are expressed as frequency (percentage). Patients were first stratified by survival status (survivors vs non-survivors) for baseline comparisons. Between-group differences were assessed using the Student *t* test or Mann–Whitney *U* test for continuous variables, and the chi-squared test for categorical variables, as appropriate. Subsequently, to analyze GV and SBPV, patients were further stratified into tertiles based on the distribution of each variability metric within each stroke subtype. The tertile cutoff values were as follows: for hemorrhagic stroke, GV: <10.69, 10.69 to 18.22, >18.22; SBPV: <9.74, 9.74 to 12.50, >12.50; for ischemic stroke, GV: <12.16, 12.16 to 21.44, >21.44; SBPV: <10.17, 10.17 to 13.14, >13.14. For analysis, the highest tertile was defined as high variability, and the lower 2 tertiles were combined and defined as low variability.

Kaplan–Meier survival analysis was performed to estimate survival curves. Univariate Cox regression was used to identify variables associated with mortality, and those with a *P* value < .05 or considered clinically significant were included in the multivariate Cox regression (Supplement Tables 1, Supplemental Digital Content 6 and 2, Supplemental Digital Content 7). The primary outcome was 28-day mortality, and the secondary outcome was 365-day mortality. To consolidate our findings, we examined the association between the number of high-variability parameters and mortality. High variability was defined as the highest tertile (T3), and low as the lower 2 tertiles (T1–T2). Subgroup analyses further evaluated the robustness of the results. In addition, we developed a variability scoring system ranging from 0 to 4 points: for both GV and SBPV, 0 points were assigned to tertile 1, 1 point to tertile 2, and 2 points to tertile 3. The combined scores were then used to calculate the VS.

Sensitivity analysis for monitoring frequency. To address the potential bias that more severely ill patients undergo more frequent monitoring, which mathematically increases the CV, we performed an additional sensitivity analysis adjusting for the total number of glucose measurements and the total number of SBP measurements per patient as continuous covariates in the Cox models.

All statistical analyses were performed using R software (version 4.3.1; R Foundation for Statistical Computing) and SPSS software (version 25.0; SPSS, Inc.).

## 3. Results

### 3.1. Baseline characteristics

This study included 2659 patients with hemorrhagic stroke and 3606 with ischemic stroke. The baseline characteristics are detailed in Table 1 (Hemorrhagic) and Table 2 (Ischemic). In brief, compared to survivors, non-survivors in both cohorts were significantly older and had a higher prevalence of comorbidities such as chronic kidney disease. Notably, non-survivors had significantly higher GV and SBPV (all *P* < .05), while they were less likely to have received antiplatelet or antihypertensive medications during their ICU stay.

**Table 1:** Baseline characteristics of patients with hemorrhagic stroke stratified by survival status.

Variable	Overall (N = 2659)	Survivors (n = 1636)	Non-survivors (n = 1023)	*P*-value
Age, yr	68.00 (56.00–79.00)	65.00 (53.00–76.00)	74.00 (63.00–83.00)	<.001
Gender, male	1361.00 (51.18%)	837.00 (51.16%)	524.00 (51.22%)	.976
Smoking history	550.00 (20.68%)	355.00 (21.70%)	195.00 (19.06%)	.102
Alcohol consumption	237.00 (8.91%)	145.00 (8.86%)	92.00 (8.99%)	.909
Vital signs				
Systolic blood pressure, mm Hg	133.00 (118.00–147.00)	132.00 (118.00–146.00)	134.00 (116.00–149.00)	.304
Diastolic blood pressure, mm Hg	73.00 (63.00–85.00)	74.00 (64.00–85.00)	71.00 (61.00–85.00)	.010
Heart rate, bpm	81.00 (71.00–93.00)	81.00 (70.00–92.00)	83.00 (72.00–95.00)	.002
Comorbidities				
Ischemic heart disease	572.00 (21.51%)	296.00 (18.09%)	276.00 (26.98%)	<.001
Hypertension	1528.00 (57.47%)	954.00 (58.31%)	574.00 (56.11%)	.263
Diabetes	646.00 (24.29%)	357.00 (21.82%)	289.00 (28.25%)	<.001
History of cerebrovascular disease	234.00 (8.80%)	115.00 (7.03%)	119.00 (11.63%)	<.001
Heart failure	386.00 (14.52%)	185.00 (11.31%)	201.00 (19.65%)	<.001
Chronic kidney disease	307.00 (11.55%)	148.00 (9.05%)	159.00 (15.54%)	<.001
Laboratory measurements				
Serum creatinine, mg/dL	0.90 (0.70–1.10)	0.80 (0.70–1.00)	0.90 (0.70–1.20)	<.001
White blood cells, 10^9^/L	10.20 (7.90–13.20)	10.20 (7.80–12.90)	10.40 (8.00–13.80)	.065
Hemoglobin, g/dL	12.10 (10.70–13.50)	12.50 (11.10–13.70)	11.70 (10.00–13.00)	<.001
Platelets, 10^9^/L	209.00 (162.00–264.00)	216.00 (170.00–265.00)	200.00 (147.00–260.00)	<.001
Total cholesterol, mg/dL	157.00 (130.00–189.00)	160.00 (133.00–192.00)	150.00 (126.00–185.00)	<.001
HDL-C, mg/dL	47.00 (37.00–58.00)	47.00 (37.00–58.00)	46.00 (35.00–57.00)	.048
LDL-C, mg/dL	84.00 (64.00–111.00)	87.00 (66.00–114.00)	79.00 (58.00–107.00)	<.001
Triglycerides, mg/dL	107.00 (74.00–153.00)	107.00 (75.00–154.00)	106.00 (73.00–152.00)	.503
Glucose, mg/dL	127.00 (106.00–156.00)	122.00 (105.00–148.00)	135.00 (110.00–169.00)	<.001
HbA1c, %	5.70 (5.30–6.40)	5.70 (5.30–6.30)	5.80 (5.40–6.50)	.005
GV, %	13.98 (8.91–21.31)	13.32 (8.18–20.00)	15.15 (10.20–24.08)	<.001
SBPV, %	11.11 (9.07–13.42)	10.75 (8.76–12.79)	11.74 (9.60–14.31)	<.001
Mean blood glucose, mg/dL	125.33 (109.00–149.00)	121.00 (107.00–140.33)	135.60 (115.33–162.33)	<.001
Mean systolic blood pressure, mm Hg	129.49 (118.94–138.20)	129.37 (119.01–138.35)	129.74 (118.21–138.06)	.474
Treatment				
Antiplatelets	913.00 (34.34%)	597.00 (36.49%)	316.00 (30.89%)	.003
Statins	1073.00 (40.35%)	693.00 (42.36%)	380.00 (37.15%)	.008
Antihypertensives	2244.00 (84.39%)	1356.00 (82.89%)	888.00 (86.80%)	.007
Antidiabetic drugs	427.00 (16.06%)	233.00 (14.24%)	194.00 (18.96%)	.001
Length of ICU stay, d	4.58 (2.47–8.88)	4.77 (2.60–9.11)	4.19 (2.26–8.38)	.044

Values are expressed as the median (interquartile range) or n (%).

GV = glycemic variability, ICU = intensive care unit, SBPV = systolic blood pressure variability.

**Table 2:** Baseline characteristics of patients with ischemic stroke stratified by survival status.

Variable	Overall (N = 3606)	Survivors (n = 1996)	Non-survivors (n = 1610)	*P*-value
Age, yr	71.00 (61.00–81.00)	68.00 (57.00–78.00)	75.00 (65.00–84.00)	<.001
Gender, male	1845.00 (51.16%)	1040.00 (52.10%)	805.00 (50.00%)	.209
Smoking history	785.00 (21.77%)	479.00 (24.00%)	306.00 (19.01%)	<.001
Alcohol consumption	286.00 (7.93%)	168.00 (8.42%)	118.00 (7.33%)	.230
Vital signs				
Systolic blood pressure, mm Hg	133.00 (114.00–151.00)	134.00 (116.00–152.00)	132.00 (112.00–150.00)	.003
Diastolic blood pressure, mm Hg	74.00 (63.00–88.00)	75.00 (64.00–89.00)	73.00 (61.00–86.00)	<.001
Heart rate, bpm	84.00 (72.00–98.00)	81.00 (70.00–95.00)	87.00 (74.00–101.00)	<.001
Comorbidities				
Ischemic heart disease	1309.00 (36.30%)	623.00 (31.21%)	686.00 (42.61%)	<.001
Hypertension	1706.00 (47.31%)	1050.00 (52.61%)	656.00 (40.75%)	<.001
Diabetes	1316.00 (36.49%)	626.00 (31.36%)	690.00 (42.86%)	<.001
History of cerebrovascular disease	992.00 (27.51%)	478.00 (23.95%)	514.00 (31.93%)	<.001
Heart failure	1068.00 (29.62%)	437.00 (21.89%)	631.00 (39.19%)	<.001
Chronic kidney disease	784.00 (21.74%)	326.00 (16.33%)	458.00 (28.45%)	<.001
Laboratory measurements				
Serum creatinine, mg/dL	1.00 (0.70–1.40)	0.90 (0.70–1.20)	1.10 (0.80–1.70)	<.001
White blood cells, 10^9^/L	10.30 (7.90–13.90)	9.90 (7.60–13.40)	10.90 (8.10–14.70)	<.001
Hemoglobin, g/dL	11.40 (9.50–13.00)	11.90 (10.10–13.40)	10.70 (9.10–12.40)	<.001
Platelets, 10^9^/L	208.00 (156.00–268.00)	209.00 (162.00–265.00)	203.00 (149.00–273.00)	.073
Total cholesterol, mg/dL	150.00 (120.00–181.00)	154.00 (125.00–185.00)	143.00 (115.00–172.00)	<.001
HDL-C, mg/dL	42.00 (33.00–54.00)	43.00 (34.00–55.00)	41.00 (32.00–53.00)	<.001
LDL-C, mg/dL	80.00 (57.00–106.00)	84.00 (61.00–111.00)	74.00 (53.00–99.00)	<.001
Triglycerides, mg/dL	111.00 (80.00–158.00)	111.00 (80.00–154.00)	111.00 (81.00–161.00)	.547
Glucose, mg/dL	126.00 (103.00–164.00)	120.00 (102.00–150.00)	135.00 (105.00–182.00)	<.001
HbA1c, %	5.80 (5.40–6.70)	5.80 (5.40–6.50)	5.90 (5.40–6.90)	<.001
GV, %	16.30 (10.27–25.14)	14.79 (8.91–22.68)	18.16 (12.12–28.78)	<.001
SBPV, %	11.54 (9.45–14.24)	11.03 (9.08–13.46)	12.20 (10.07–15.17)	<.001
Mean blood glucose, mg/dL	125.67 (107.40–155.57)	120.67 (104.10–144.45)	134.29 (112.00–170.67)	<.001
Mean systolic blood pressure, mm Hg	127.54 (114.39–141.03)	129.18 (116.00–142.66)	125.50 (112.25–138.93)	<.001
Treatment				
Antiplatelets	2656.00 (73.66%)	1544.00 (77.35%)	1112.00 (69.07%)	<.001
Statins	2535.00 (70.30%)	1481.00 (74.20%)	1054.00 (65.47%)	<.001
Antihypertensives	3052.00 (84.64%)	1645.00 (82.41%)	1407.00 (87.39%)	<.001
Antidiabetic drugs	912.00 (25.29%)	452.00 (22.65%)	460.00 (28.57%)	<.001
Length of ICU stay, d	3.74 (2.03–7.01)	3.37 (1.90–6.37)	3.99 (2.35–7.89)	<.001

Values are expressed as the median (interquartile range) or n (%).

GV = glycemic variability, ICU = intensive care unit, SBPV = systolic blood pressure variability.

### 3.2. The correlation between GV and SBPV in patients with hemorrhagic and ischemic stroke

GV and SBPV demonstrated significant but weak positive correlations in both hemorrhagic (*R* = 0.145) and ischemic (*R* = 0.058) stroke (*P* < .001 for both; Supplementary Figs. 2, Supplemental Digital Content 2 and 3, Supplemental Digital Content 3), suggesting shared yet distinct underlying mechanisms that influence prognosis.

### 3.3. Individual and combined effects of glycemic and blood pressure variability (BPV) on mortality

Both GV and SBPV demonstrated significant individual and additive associations with mortality in stroke patients.

#### 3.3.1. Individual effects of GV and SBPV

Kaplan–Meier survival curves revealed a stepwise decrease in survival probability across ascending tertiles of both GV and SBPV for 28-day and 1-year mortality (Supplementary Figs. 4, Supplemental Digital Content 4 and 5, Supplemental Digital Content 5). In fully adjusted Cox regression models, this graded relationship was confirmed. Among patients with hemorrhagic stroke, those in the highest GV tertile had a 1.8-fold higher risk of 28-day mortality (hazard ratio [HR] = 1.84, 95% confidence interval [CI] 1.38–2.44) and a 1.6-fold higher risk of 1-year mortality (HR = 1.62, 95% CI 1.26–2.09) compared to those in the lowest tertile. Similarly, the highest SBPV tertile was associated with significantly increased mortality (e.g., 28-day mortality HR = 1.67, 95% CI 1.25–2.22). A consistent pattern was observed in ischemic stroke patients, where the highest GV and SBPV tertiles were independently associated with approximately a 1.4- and 1.6-fold increased risk of mortality, respectively (all findings detailed in Supplementary Tables 3, Supplemental Digital Content 8 and 4, Supplemental Digital Content 9).

#### 3.3.2. Additive combined effect of high GV and high SBPV

**Figure 1.**
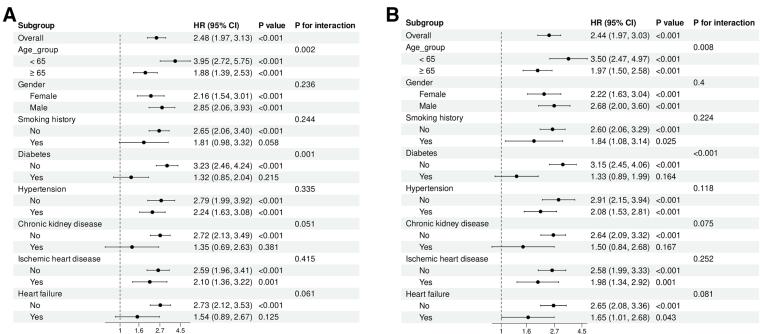
Subgroup analyses of the association between the number of high-variability parameters and mortality in hemorrhagic stroke patients. Forest plots display hazard ratios for (A) 28-day and (B) 365-day mortality. The number of high-variability parameters (0, 1, or 2) refers to the presence of high glycemic variability (GV) and/or high systolic blood pressure variability (SBPV). Analyses were adjusted for the covariates listed in Table 3. CI = confidence interval, HR = hazard ratio.

The combination of high GV and high SBPV was associated with the most significant mortality risk. As summarized in Table 3, hemorrhagic stroke patients exhibiting both high parameters faced a 2.5-fold higher risk of 28-day mortality (HR = 2.51, 95% CI 1.89–3.33) and a 2.3-fold higher risk of 1-year mortality (HR = 2.25, 95% CI 1.74–2.91) after full adjustment, compared to those with both low variability. Similarly, in ischemic stroke, the combination doubled the risk for both 28-day (HR = 2.01, 95% CI 1.57–2.58) and 1-year mortality (HR = 1.99, 95% CI 1.59–2.49; Table 4). Kaplan–Meier curves visually confirmed these additive effects, with the worst survival consistently observed in the high-GV/high-SBPV group (Fig. 1).

**Table 3:** Glycemic and blood pressure variability: combined impact on mortality in hemorrhagic stroke.

	Low GV low SBPV (N = 1233)	High GV low SBPV (N = 539)	Low GV high SBPV (N = 539)	High GV high SBPV (N = 348)
28-d mortality				
Model 1	Ref	1.542 (1.159–2.051) *P* = .003	1.872 (1.426–2.457) *P* < .001	3.279 (2.504–4.294) *P* < .001
Model 2	Ref	1.498 (1.126–1.994) *P* = .006	1.700 (1.292–2.238) *P* < .001	3.068 (2.340–4.023) *P* < .001
Model 3	Ref	1.227 (0.904–1.666) *P* = .190	1.800 (1.365–2.375) *P* < .001	2.510 (1.890–3.333) *P* < .001
365-d mortality				
Model 1	Ref	1.356 (1.044–1.759) *P* = .022	1.615 (1.259–2.072) *P* < .001	2.983 (2.338–3.806) *P* < .001
Model 2	Ref	1.317 (1.015–1.709) *P* = .039	1.484 (1.154–1.909) *P* = .002	2.815 (2.203–3.596) *P* < .001
Model 3	Ref	1.060 (0.803–1.400) *P* = .680	1.525 (1.183–1.965) *P* = .001	2.250 (1.739–2.909) *P* < .001

Model 1 was unadjusted. Model 2 adjusted for sex and age. Model 3 adjusted for age, sex, smoking history, alcohol consumption, heart rate, ischemic heart disease, diabetes, heart failure, hemoglobin, platelets, white blood cells, total cholesterol, HDL-C, LDL-C, triglycerides, serum creatinine, glucose, as well as the use of antiplatelet agents and statins.

GV = glycemic variability, SBPV = systolic blood pressure variability.

**Table 4:** Glycemic and blood pressure variability: combined impact on mortality in ischemic stroke.

	Low GV low SBPV (N = 1668)	High GV low SBPV (N = 736)	Low GV high SBPV (N = 736)	High GV high SBPV (N = 466)
28-d mortality				
Model 1	Ref	1.393 (1.100–1.765) *P* = .006	1.827 (1.465–2.277) *P* < .001	2.470 (1.956–3.119) *P* < .001
Model 2	Ref	1.344 (1.061–1.703) *P* = .014	1.704 (1.366–2.127) *P* < .001	2.374 (1.879–3.000) *P* < .001
Model 3	Ref	1.127 (0.880–1.443) *P* = .345	1.571 (1.254–1.968) *P* < .001	2.011 (1.567–2.581) *P* < .001
365-d mortality				
Model 1	Ref	1.394 (1.128–1.723) *P* = .002	1.789 (1.467–2.182) *P* < .001	2.532 (2.057–3.118) *P* < .001
Model 2	Ref	1.356 (1.097–1.676) *P* = .005	1.699 (1.391–2.075) *P* < .001	2.462 (1.998–3.035) *P* < .001
Model 3	Ref	1.113 (0.892–1.389) *P* = .343	1.524 (1.243–1.868) *P* < .001	1.991 (1.593–2.488) *P* < .001

Model 1 was unadjusted. Model 2 adjusted for sex and age. Model 3 adjusted for age, sex, hemoglobin, white blood cells, HDL-C, LDL-C, total cholesterol, serum creatinine, heart rate, history of cerebrovascular disease, heart failure, ischemic heart disease, statin use, antiplatelet use, glucose, and systolic blood pressure.

GV = glycemic variability, SBPV = systolic blood pressure variability.

#### 3.3.3. Sensitivity analysis

To address potential bias from using only the first 72 hours of ICU data, we conducted a sensitivity analysis incorporating all blood glucose and SBP measurements from the entire ICU stay. The results of this analysis remained consistent with our primary findings, confirming the robustness of the association between GV, SBPV, and mortality (Supplement Tables 5, Supplemental Digital Content 10 and 6, Supplemental Digital Content 11).

Additional sensitivity analysis for monitoring frequency. After additional adjustment for monitoring frequency (number of glucose and SBP measurements), the association between high combined variability (both high GV and high SBPV) and 28-day mortality remained statistically significant but was attenuated: adjusted HR changed from 2.51 (95% CI 1.89–3.33) to 2.01 (95% CI 1.55–2.61) in hemorrhagic stroke, and from 2.01 (1.57–2.58) to 1.71 (1.34–2.19) in ischemic stroke. This indicates that monitoring intensity indeed contributes to overestimation, but a substantial independent association persists.

### 3.4. Relationship between the number and VSs of highly variable parameters and mortality rates in patients with hemorrhagic and ischemic strokes

To further quantify the cumulative risk, we first evaluated the number of high-variability parameters (GV or SBPV). A graded increase in mortality was observed with each additional high-variability parameter (*P* for trend < .05; Supplementary Tables 7, Supplemental Digital Content 12 and 8, Supplemental Digital Content 13). For example, hemorrhagic stroke patients with both high GV and high SBPV had a 157% higher risk of 28-day mortality (odds ratio = 2.572, 95% CI 1.939–3.411) compared to those with neither.

**Figure 2.**
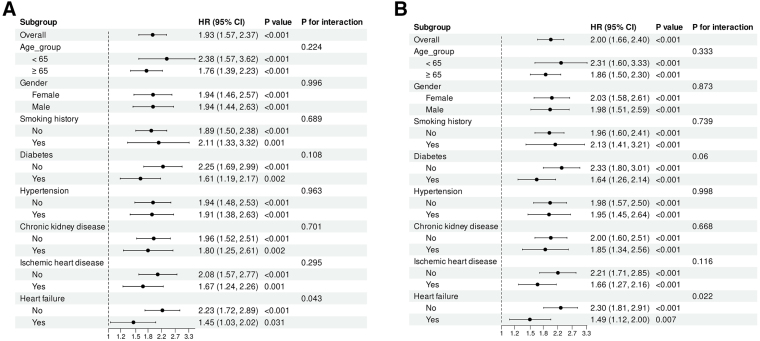
Subgroup analyses of the association between the number of high-variability parameters and mortality in ischemic stroke patients. Forest plots display hazard ratios for (A) 28-day and (B) 365-day mortality. The number of high-variability parameters (0, 1, or 2) refers to the presence of high glycemic variability (GV) and/or high systolic blood pressure variability (SBPV). Analyses were adjusted for the covariates listed in Table 4. CI = confidence interval, HR = hazard ratio.

We subsequently integrated GV and SBPV into a composite VS (range 0–4). This score revealed a strong, graded association with mortality across both stroke types. For hemorrhagic stroke, the 28-day mortality rate climbed from 7.78% (VS = 0) to 27.30% (VS = 4), while the 1-year mortality rate increased from 10.37 to 31.90%. In ischemic stroke, the rates rose from 9.01 to 24.68% for 28-day mortality, and from 11.16 to 31.12% for 1-year mortality (Figs. 1 and 2).

**Figure 3.**
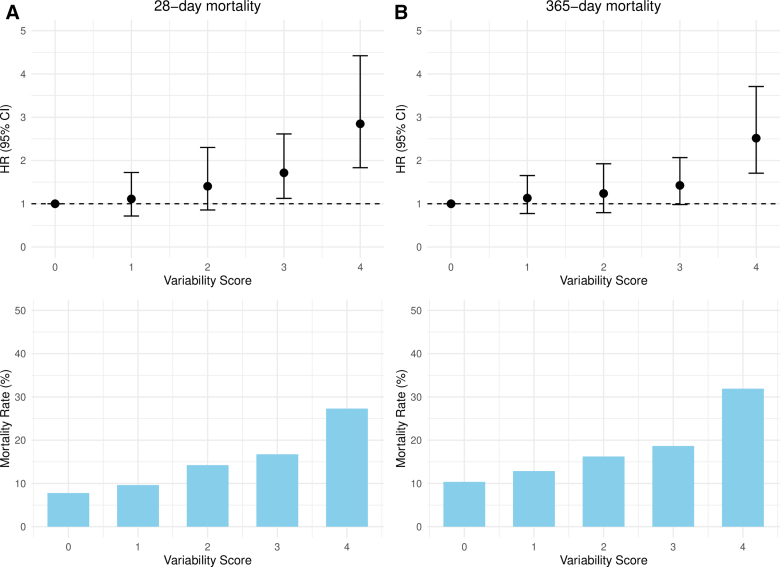
Association between the composite variability score and mortality in patients with hemorrhagic stroke. Panels show the association with (A) 28-day and (B) 365-day mortality. Each panel presents adjusted hazard ratios (HRs) with 95% confidence intervals (CIs) from multivariable Cox regression and actual mortality rates across variability scores. The variability score (range 0–4) was calculated by summing the points assigned to GV and SBPV tertiles (Tertile 1 = 0 points, Tertile 2 = 1 point, Tertile 3 = 2 points). Multivariable models were adjusted for covariates listed in Table 3. GV = glycemic variability, SBPV = systolic blood pressure variability.

**Figure 4.**
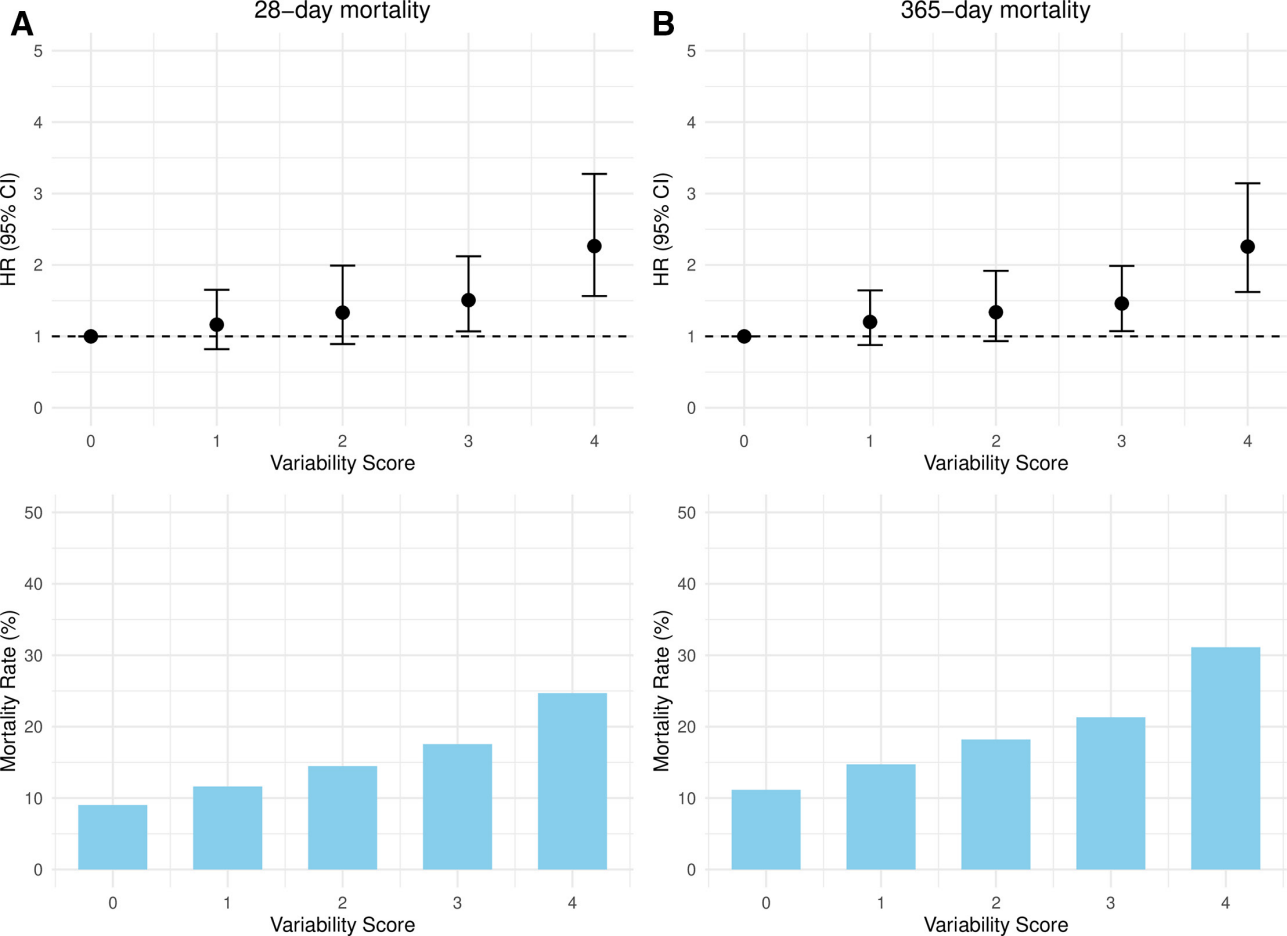
Association between the composite variability score and mortality in patients with ischemic stroke. Panels show the association with (A) 28-day and (B) 365-day mortality. Each panel presents adjusted hazard ratios (HRs) with 95% confidence intervals (CIs) from multivariable Cox regression and actual mortality rates across variability scores. The variability score (range 0–4) was calculated by summing the points assigned to GV and SBPV tertiles (Tertile 1 = 0 points, Tertile 2 = 1 point, Tertile 3 = 2 points). Multivariable models were adjusted for covariates listed in Table 4. GV = glycemic variability, SBPV = systolic blood pressure variability.

This dose–response relationship was confirmed in multivariable analysis. With each point increase in the VS, the HRs for mortality rose progressively. In hemorrhagic stroke, the adjusted HR for 28-day mortality increased from 1.112 (95% CI 0.717–1.723) at VS = 1 to 2.848 (95% CI 1.834–4.422) at VS = 4. A similar graded increase was observed for 1-year mortality and in the ischemic stroke cohort (Figs. 3 and 4).

## 4. Discussion

In this large retrospective cohort of critically ill stroke patients, our study provides compelling evidence that short-term GV and SBPV during the initial ICU stay are not only independent but also additive predictors of both 28-day and 1-year mortality. A key strength of our findings is the clear dose–response relationship observed, in which mortality risk increased progressively with both the number of high-variability parameters and the composite VS. Crucially, these robust associations persisted after comprehensive adjustment for mean glucose levels, mean blood pressure, and other potential confounders. This underscores that the fluctuations in these parameters, distinct from their absolute values, carry unique and critical prognostic value in the acute phase of stroke.

Our study achieves academic breakthroughs in 3 key areas: it is the first to unveil the unique clinical significance of the “acute-phase synergistic effect” between glycemic and BPV in neurocritical care; it establishes a composite scoring model through the innovative integration of these 2 parameters; and it translates complex physiological dysregulation into an immediate risk assessment tool applicable at the bedside. First, regarding the clinical context, while prior studies have established GV and SBPV as independent risk factors for adverse outcomes across various populations,^[4,6,7]^ the majority have focused on long-term, visit-to-visit variability in ambulatory or chronic disease settings.^[11–13]^ Our study decisively shifts this focus to the acute, in-ICU phase, a period where the injured brain is exquisitely vulnerable to systemic physiological fluctuations. Second, methodologically, by integrating both parameters, we move beyond the isolated examination common in prior studies of acute illness, which have typically investigated either GV^[16–18]^ or SBPV^[19–25]^ in isolation. We are among the first to quantitatively demonstrate their synergistic, additive effect on mortality in this vulnerable neurocritical care population. Finally, in terms of clinical application, the novel composite VS translates complex physiological dysregulation into a practical tool, offering a more holistic and immediate risk assessment model at the bedside.

The mechanisms underlying the synergistic detriment of GV and SBPV are likely multifaceted and self-reinforcing. We hypothesize that acute glycemic fluctuations induce oxidative stress, mitochondrial dysfunction, and cellular susceptibility, particularly in endothelial cells.^[26–29]^ When the metabolic stress induced by acute glycemic fluctuations (the first hit) combines with the direct mechanical shear stress exerted on the vascular endothelium by abrupt blood pressure fluctuations (the second hit), their synergistic effect may lead to accelerated endothelial dysfunction, further impaired cerebral autoregulation, and increased blood-brain barrier disruption.^[30,31]^ This creates a vicious cycle: endothelial damage promotes inflammation and thrombosis, which, in turn, can worsen both glycemic dysregulation and blood pressure lability.^[32]^ This pathophysiological synergy provides a coherent explanation for our central finding of a markedly higher mortality when both variabilities are present, as the combined insult likely leads to more severe secondary brain injury.

Our results are consistent with, yet significantly advance, the existing literature on variability in acute illness. GV has been correlated with high mortality in critically ill patients^[16,17]^ and identified as an independent predictor of mortality in acute stroke.^[18]^ Similarly, higher BPV in patients with intracerebral hemorrhage and acute cerebral infarction correlates with increased mortality and poorer functional outcomes.^[19–25]^ A retrospective analysis of the MIMIC-III database also found that higher SBPV within 72 hours of admission was independently associated with in-hospitalmortality.^[7]^ However, by integrating both parameters and demonstrating their additive effect through a novel scoring system, our study provides a more comprehensive risk assessment model that moves beyond examining these factors in isolation. The graded increase in risk with higher VSs offers a powerful tool for quantifying cumulative physiological dysregulation.

Although the observational nature of our study precludes definitive causal inference, the robustness of the observed associations and their clear dose–response relationship, coupled with a plausible biological mechanism, strongly suggest that glycemic and BPV are not merely clinical indicators of disease severity but may themselves be potential therapeutic targets. The clinical implications of our study are substantial. First, monitoring GV and SBPV could significantly enhance risk stratification in the neuro-ICU. The simple VS we proposed provides clinicians with a practical method for identifying a high-risk subgroup of stroke patients who may benefit from more intensive monitoring and personalized management strategies. This score effectively encapsulates the cumulative physiological stress imposed by concurrent metabolic and hemodynamic instability, providing a quantifiable measure that is readily obtainable from routine ICU monitoring data. Second, our findings suggest that the therapeutic goal in acute stroke should expand beyond merely controlling average levels of glucose and blood pressure to include stabilizing their fluctuations. This represents a paradigm shift in management thinking. Several trials indicate that specific antidiabetic and antihypertensive agents may differentially impact variability. For example, glucagon-like peptide-1 receptor agonists^[32,33]^ and sodium-glucose cotransporter-2 inhibitors^[34]^ have been shown to lower GV. At the same time, calcium channel blockers and thiazide diuretics may reduce SBPV more effectively than other drug classes.^[35,36]^ Consequently, our study directs future interventional research to a critical question: Can therapies specifically selected to mitigate GV and SBPV translate into improved survival and functional outcomes for stroke patients? Future studies should prioritize validating this VS in independent, multi-center cohorts. Furthermore, interventional trials are warranted to investigate whether a “variability-targeted” management protocol, potentially leveraging the differential effects of specific antihypertensive and antidiabetic agents, can improve outcomes compared to standard care focused solely on absolute values.

Toward a variability-targeted protocol. Based on our findings, we propose a two-step framework for future interventional trials. First, risk stratification – upon ICU admission, calculate GV and SBPV from the first 24 to 72 hours of ICU data. We propose that a composite score ≥ 3 should alert clinicians to a high-risk phenotype and prompt more intensive monitoring. MIMIC-IV-derived evidence has confirmed that 24-hour SBPV independently predicts 28-day mortality.^[37]^ Second, stabilization strategy – for GV, use protocolized insulin with a target glucose range of 140 to 180 mg/dL and a gradual correction (e.g., targeting a decline of ≤ 50 mg/dL per hour, a commonly adopted safety threshold in clinical practice) as recommended by the 2024 SCCM guidelines for conventional glucose control in critically ill adults^[38]^; consider continuous glucose monitoring to detect early swings. For SBPV, use titratable intravenous agents (e.g., nicardipine) with a target SBP < 160 mm Hg, avoiding levels < 130 mm Hg as suggested by the Chinese consensus statement on hypertension management in stroke patients,^[39]^ and avoid intermittent bolus doses to minimize BP oscillations. A “variability dashboard” that displays real-time CV over the past 6 hours could guide bedside decisions. This represents a paradigm shift from merely controlling mean values to actively stabilizing physiological fluctuations.

Clinical implications: our findings offer several actionable insights for bedside practice. First, the composite VS (range 0–4) can be readily calculated from routine ICU monitoring data within the first 72 hours. We propose that a score ≥ 3 should alert clinicians to a high-risk phenotype, prompting more intensive neurological monitoring and stricter avoidance of iatrogenic glucose/BP swings. Second, in patients with high GV, insulin infusion protocols should aim for gradual correction (e.g., glucose decline ≤ 50 mg/dL per hour) rather than rapid normalization; continuous glucose monitoring may help detect and prevent abrupt fluctuations. Third, for high SBPV, prefer intravenous antihypertensive agents with short half-lives (e.g., nicardipine over labetalol) and avoid bolus dosing to minimize BP oscillations. These practical strategies, while awaiting prospective validation, represent a shift from targeting mean values to promoting physiological stability.

### 4.1. Limitations

Several limitations of our study should be acknowledged. First, as a retrospective analysis of a single-center database, large unmeasured confounding factors may persist despite our multivariate adjustments. Second, the timing and frequency of glucose and blood pressure measurements were based on clinical practice rather than a rigid protocol, which could introduce measurement bias. Patients with greater illness severity inherently underwent more frequent checks, which could artificially elevate the CV and bias estimates upward. Our sensitivity analysis adjusting for measurement counts confirmed that while some overestimation exists, the independent association remained significant. Nonetheless, the defined variability thresholds may not be directly generalizable to settings with lower monitoring density, such as general wards or stroke units without continuous monitoring. Third, the MIMIC-IV database lacks detailed information on the causes of death, preventing us from exploring the specific mechanisms of mortality. Finally, our findings derive from a single-center tertiary ICU database with intensive monitoring protocols. The variability thresholds and risk scores may not directly generalize to general wards, stroke units, or healthcare systems with different monitoring practices. External validation in diverse clinical settings is required before clinical application.

## 5. Conclusion

In conclusion, this study establishes that short-term GV and SBPV during ICU admission are not only independent but also additive predictors of mortality in critically ill stroke patients. The risk of death exhibits a strong, graded relationship with a composite VS, highlighting the cumulative burden of physiological dysregulation. These findings support incorporating variability assessment into routine clinical practice to enhance prognostication. Ultimately, targeting the stability of metabolic parameters, as well to their absolute values, may open new avenues for therapeutic interventions aimed at improving outcomes in this vulnerable population.

## Acknowledgments

We would like to thank all participants in this study. The authors also acknowledge the contributions of the MIMIC-IV database.

## Author contributions

**Conceptualization:** Chanchan Miao, Xuejun Gao.

**Formal analysis:** Chanchan Miao, Lele Kang.

**Data curation:** Lele Kang.

**Investigation:** Lele Kang.

**Visualization:** Lele Kang.

**Methodology:** Lele Kang.

**Supervision:** Xuejun Gao.

**Project administration:** Xuejun Gao.

**Writing – original draft:** Chanchan Miao.

**Writing – review & editing:** Chanchan Miao, Xuejun Gao.


























