# Supplementary figures and images for: Synergistic mortality risk of glycemic and blood pressure variability in critical stroke: A retrospective cohort study from the MIMIC-IV database
Source: Medicine (Baltimore). 2026 Jun 26;105(26):e49291. doi: 10.1097/MD.0000000000049291 (PMC13313635; doi:10.1097/MD.0000000000049291)

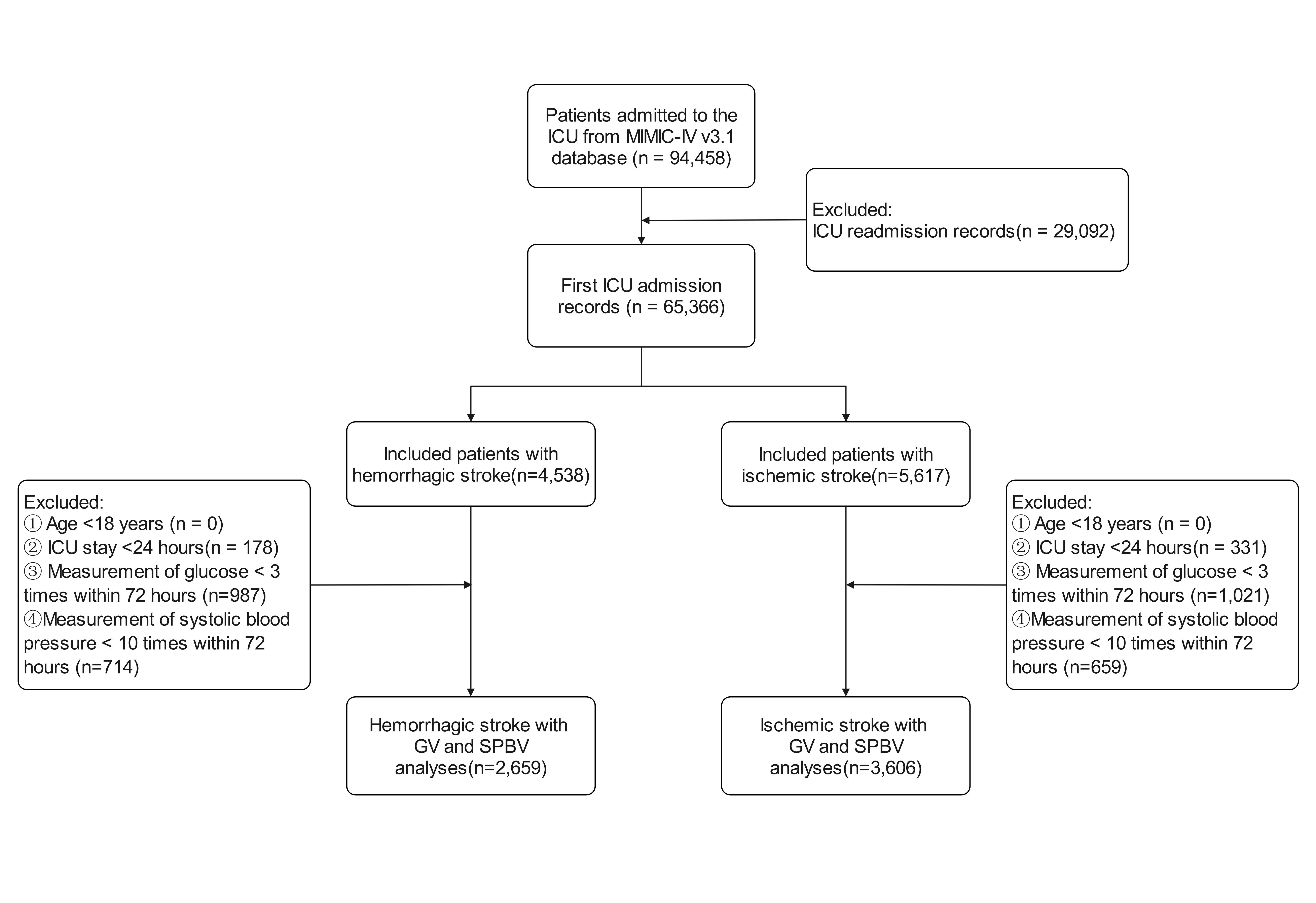

Supplement: Supplementary file 1 [file medi-105-e49291-s001.tif]

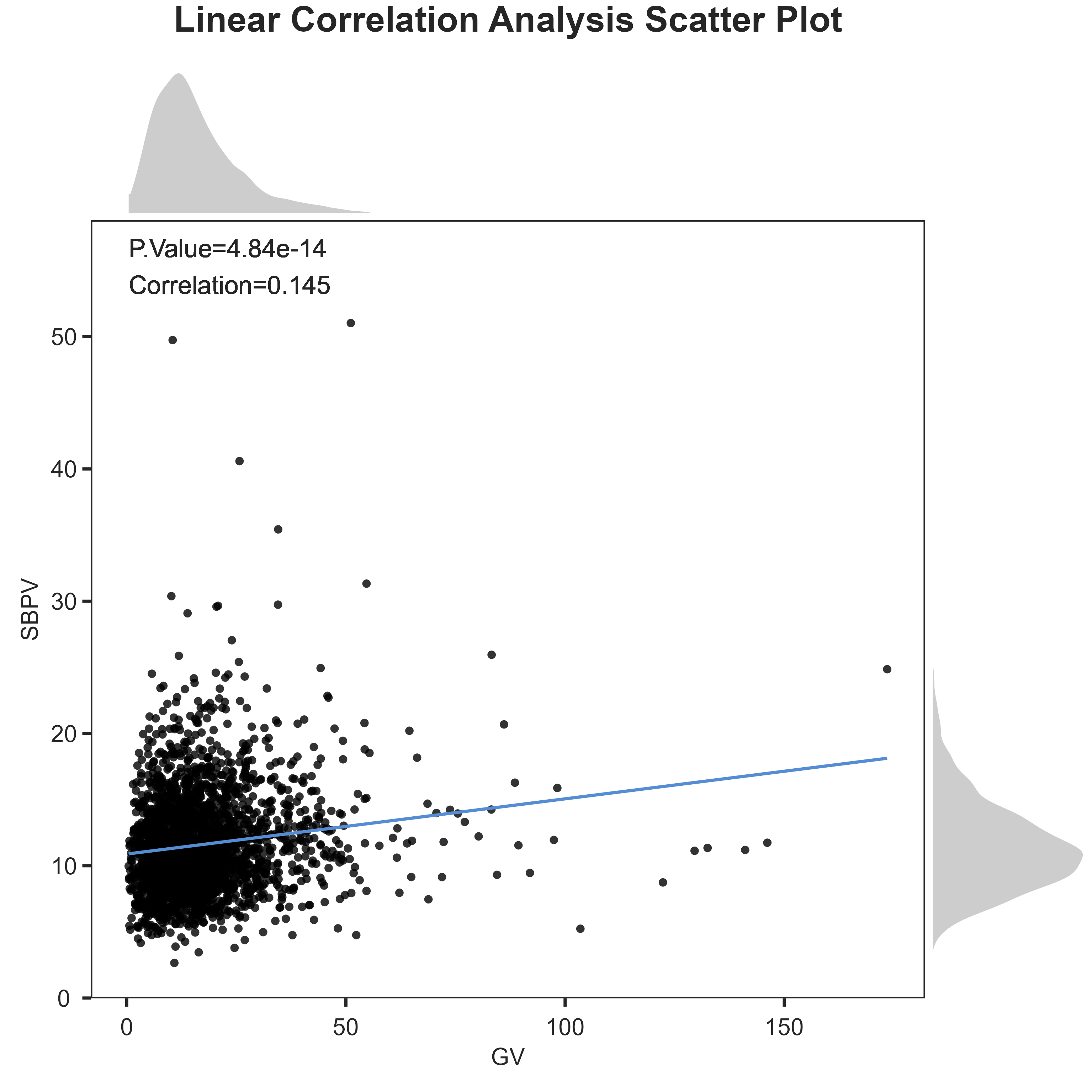

Supplement: Supplementary file 2 [file medi-105-e49291-s002.tif]

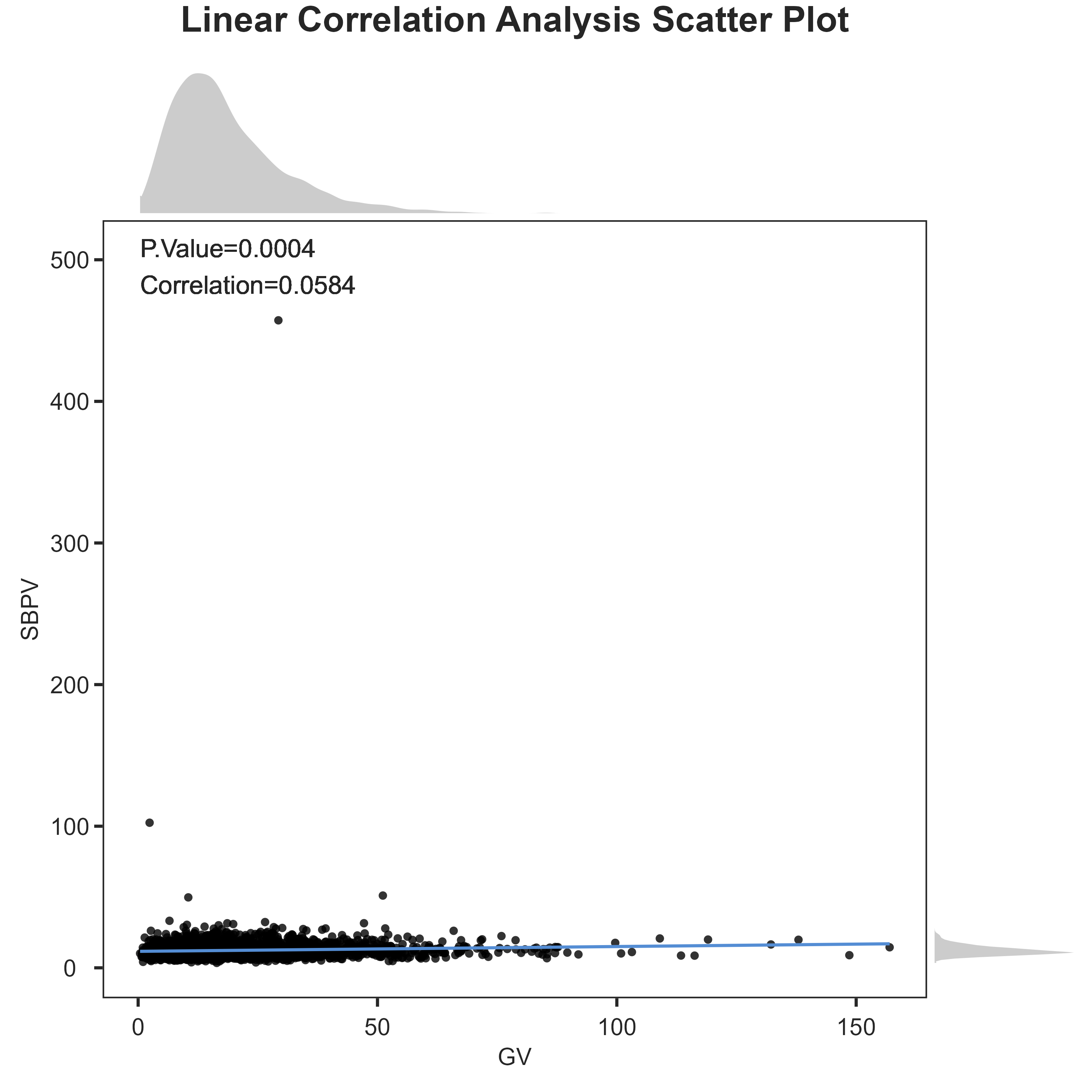

Supplement: Supplementary file 3 [file medi-105-e49291-s003.tif]

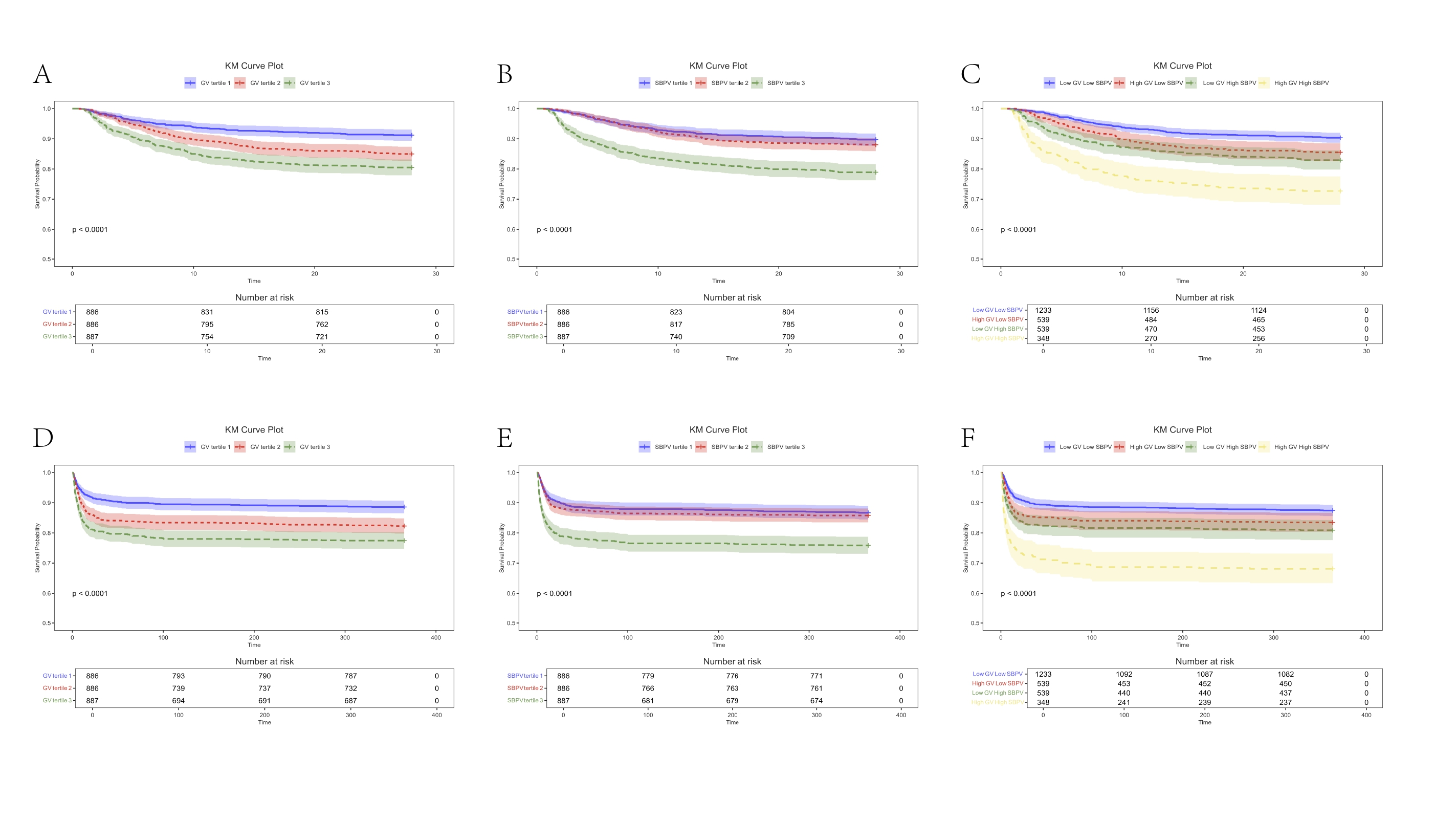

Supplement: Supplementary file 4 [file medi-105-e49291-s004.tif]

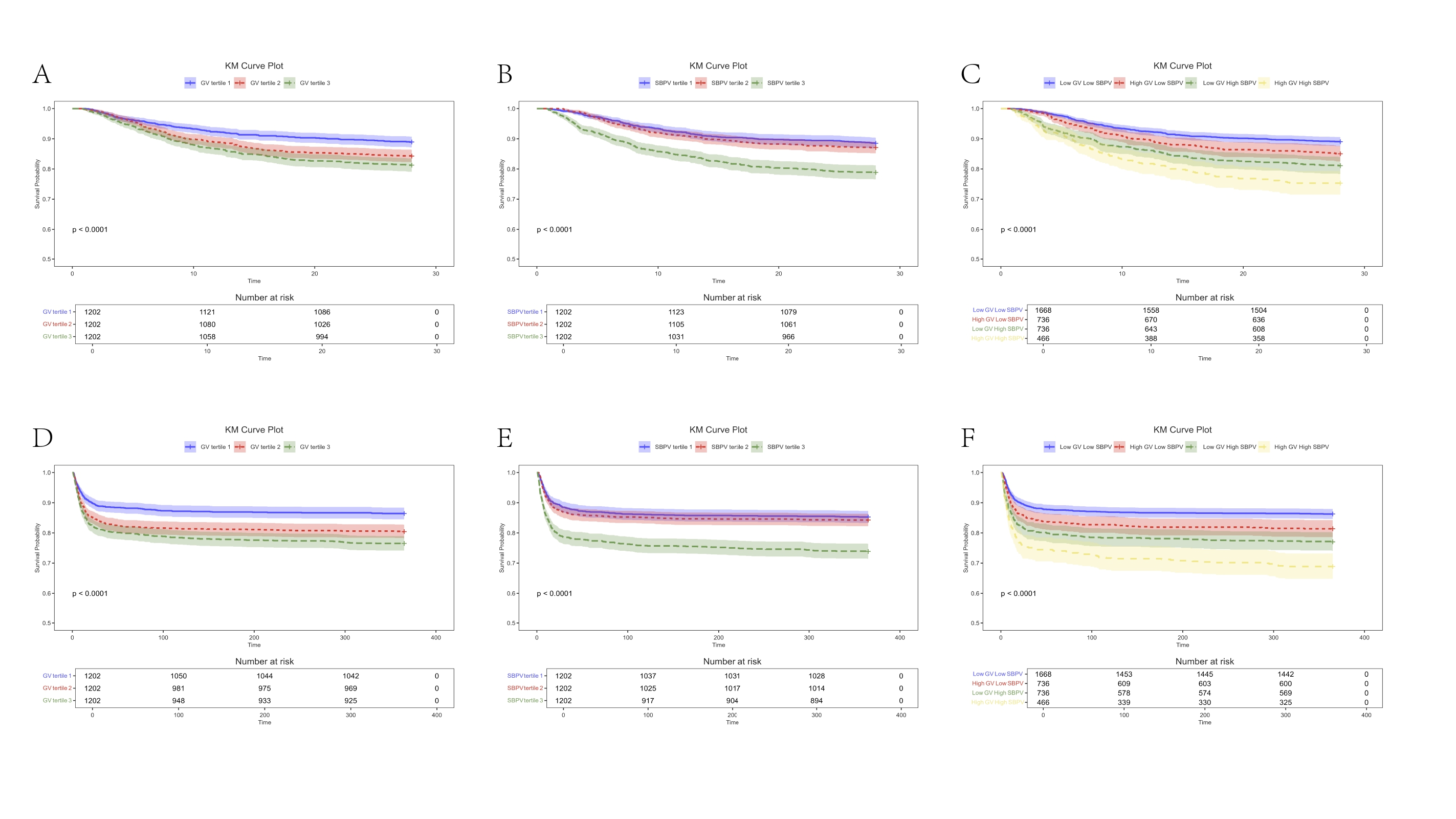

Supplement: Supplementary file 5 [file medi-105-e49291-s005.tif]
