# Supplementary material for: Synergistic mortality risk of glycemic and blood pressure variability in critical stroke: A retrospective cohort study from the MIMIC-IV database
Source: Medicine (Baltimore). 2026 Jun 26;105(26):e49291. doi: 10.1097/MD.0000000000049291 (PMC13313635; doi:10.1097/MD.0000000000049291)
Supplement: Supplementary file 6 [file medi-105-e49291-s006.docx]

**Supplement Table 1. Univariate Cox regression analysis of hemorrhagic stroke patients**

| **Variable** | **non-survivors (n = 1023)** | **p-value** |
| --- | --- | --- |
| Age, years | 1.019 (1.012–1.026) | P<0.001 |
| Gender, male | 0.953 (0.780–1.164) | P=0.635 |
| Smoking history | 0.727 (0.555–0.952) | P=0.021 |
| Alcohol consumption | 1.413 (1.036–1.929) | P=0.029 |
| **Vital signs** |  |  |
| Systolic blood pressure, mmHg | 0.997 (0.992–1.001) | P=0.148 |
| Diastolic blood pressure, mmHg | 0.995 (0.989–1.001) | P=0.075 |
| Heart rate, bpm | 1.008 (1.002–1.014) | P=0.004 |
| **Comorbidities** |  |  |
| Ischemic heart disease | 1.352 (1.078–1.695) | P=0.009 |
| Hypertension | 0.930 (0.760–1.138) | P=0.481 |
| Diabetes | 1.309 (1.051–1.630) | P=0.016 |
| History of cerebrovascular disease | 0.787 (0.536–1.157) | P=0.224 |
| Heart failure | 1.489 (1.158–1.913) | P=0.002 |
| Chronic kidney disease | 1.183 (0.881–1.588) | P=0.264 |
| **Laboratory measurements** |  |  |
| Serum creatinine, mg/dL | 1.073 (1.039–1.108) | P<0.001 |
| White blood cells, 10⁹/L | 1.008 (1.005–1.012) | P<0.001 |
| Hemoglobin, g/dL | 0.922 (0.880–0.966) | P=0.001 |
| Platelets, 10⁹/L | 0.997 (0.996–0.998) | P<0.001 |
| Total cholesterol, mg/dL | 0.996 (0.994–0.998) | P=0.001 |
| HDL-C, mg/dL | 0.992 (0.986–0.998) | P=0.006 |
| LDL-C, mg/dL | 0.996 (0.993–0.999) | P=0.005 |
| Triglycerides, mg/dL | 1.001 (1.000–1.001) | P<0.001 |
| Glucose, mg/dL | 1.004 (1.003–1.005) | P<0.001 |
| HbA1c, % | 1.063 (0.999–1.131) | P=0.052 |
| GV, % | 1.017 (1.012–1.021) | P<0.001 |
| SBPV, % | 1.083 (1.066–1.100) | P<0.001 |
| **Treatment** |  |  |
| Antiplatelets | 0.466 (0.364–0.596) | P<0.001 |
| Statins | 0.450 (0.356–0.567) | P<0.001 |
| Antihypertensives | 0.936 (0.712–1.230) | P=0.634 |
| Antidiabetic drugs | 1.153 (0.891–1.492) | P=0.279 |
