# Supplementary material for: Synergistic mortality risk of glycemic and blood pressure variability in critical stroke: A retrospective cohort study from the MIMIC-IV database
Source: Medicine (Baltimore). 2026 Jun 26;105(26):e49291. doi: 10.1097/MD.0000000000049291 (PMC13313635; doi:10.1097/MD.0000000000049291)
Supplement: Supplementary file 7 [file medi-105-e49291-s007.docx]

**Supplement Table 2. Univariate Cox regression analysis of ischemic stroke patients**

| **Variable** | **non-survivors (n = 1610)** | **p-value** |
| --- | --- | --- |
| Age, years | 1.020 (1.014–1.027) | P<0.001 |
| Gender, male | 1.011 (0.855–1.195) | P=0.901 |
| Smoking history | 0.972 (0.792–1.193) | P=0.786 |
| Alcohol consumption | 1.202 (0.901–1.603) | P=0.212 |
| **Vital signs** |  |  |
| Systolic blood pressure, mmHg | 0.996 (0.993–0.999) | P=0.012 |
| Diastolic blood pressure, mmHg | 0.998 (0.993–1.002) | P=0.291 |
| Heart rate, bpm | 1.014 (1.010–1.018) | P<0.001 |
| **Comorbidities** |  |  |
| Ischemic heart disease | 1.341 (1.132–1.589) | P=0.001 |
| Hypertension | 0.895 (0.756–1.060) | P=0.198 |
| Diabetes | 1.164 (0.981–1.381) | P=0.082 |
| History of cerebrovascular disease | 0.754 (0.617–0.921) | P=0.006 |
| Heart failure | 1.564 (1.317–1.858) | P<0.001 |
| Chronic kidney disease | 1.187 (0.977–1.441) | P=0.085 |
| **Laboratory measurements** |  |  |
| Serum creatinine, mg/dL | 1.068 (1.031–1.105) | P<0.001 |
| White blood cells, 10⁹/L | 1.010 (1.007–1.014) | P<0.001 |
| Hemoglobin, g/dL | 0.952 (0.920–0.986) | P=0.006 |
| Platelets, 10⁹/L | 1.000 (0.999–1.001) | P=0.960 |
| Total cholesterol, mg/dL | 0.997 (0.995–0.999) | P=0.001 |
| HDL-C, mg/dL | 0.993 (0.988–0.998) | P=0.009 |
| LDL-C, mg/dL | 0.996 (0.994–0.999) | P=0.001 |
| Triglycerides, mg/dL | 1.000 (1.000–1.001) | P=0.053 |
| Glucose, mg/dL | 1.002 (1.002–1.003) | P<0.001 |
| HbA1c, % | 1.033 (0.983–1.085) | P=0.203 |
| GV, % | 1.011 (1.006–1.016) | P<0.001 |
| SBPV, % | 1.005 (1.002–1.009) | P=0.001 |
| **Treatment** |  |  |
| Antiplatelets | 0.512 (0.432–0.608) | P<0.001 |
| Statins | 0.456 (0.385–0.540) | P<0.001 |
| Antihypertensives | 1.082 (0.850–1.377) | P=0.522 |
| Antidiabetic drugs | 0.843 (0.690–1.029) | P=0.093 |
