# Supplementary material for: Synergistic mortality risk of glycemic and blood pressure variability in critical stroke: A retrospective cohort study from the MIMIC-IV database
Source: Medicine (Baltimore). 2026 Jun 26;105(26):e49291. doi: 10.1097/MD.0000000000049291 (PMC13313635; doi:10.1097/MD.0000000000049291)
Supplement: Supplementary file 8 [file medi-105-e49291-s008.docx]

**Supplement Table 3. Cox regression and trend test for hemorrhagic stroke**

|  |  | **Model 1** | **Model 2** | **Model 3** |
| --- | --- | --- | --- | --- |
| **28-day mortality** | **GV** | 1.017 (1.012–1.021) P<0.001 | 1.017 (1.012–1.021) P<0.001 | 1.007 (1.001–1.014) P=0.031 |
|  | GV tertiles | P for trend < 0.001 | P for trend < 0.001 | P for trend < 0.001 |
|  | Tertile 1, n = 886 | Ref | Ref | Ref |
|  | Tertile 2, n = 886 | 1.751 (1.324–2.316) P<0.001 | \| 1.710(1.293–2.262)  P<0.001 \| 1.293 \| 2.262 \| 0 \| \| --- \| --- \| --- \| --- \| \| 1.77 \| 3.022 \| 0 \| | 1.591 (1.199–2.112) P=0.001 |
|  | Tertile 3, n = 887 | 2.375 (1.818–3.103) P<0.001 | 2.313 (1.770–3.022) P<0.001 | 1.837 (1.381–2.443) P<0.001 |
|  | **SBPV** | 1.083 (1.066–1.100) P<0.001 | 1.081 (1.063–1.099) P<0.001 | 1.081 (1.061–1.100) P<0.001 |
|  | SBPV tertiles | P for trend < 0.001 | P for trend < 0.001 | P for trend < 0.001 |
|  | Tertile 1, n = 886 | Ref | Ref | Ref |
|  | Tertile 2, n = 886 | 1.170 (0.884–1.549) P=0.271 | 1.064 (0.801–1.412) P=0.669 | 0.945 (0.709–1.260) P=0.700 |
|  | Tertile 3, n = 887 | 2.235 (1.740–2.872) P<0.001 | 1.977 (1.530–2.554) P<0.001 | 1.898 (1.467–2.457) P<0.001 |
| **365-day mortality** | **GV** | 1.016 (1.012–1.020) P<0.001 | 1.016 (1.012–1.020) P<0.001 | 1.007 (1.001–1.014) P=0.020 |
|  | GV tertiles | P for trend < 0.001 | P for trend < 0.001 | P for trend < 0.001 |
|  | Tertile 1, n = 886 | Ref | Ref | Ref |
|  | Tertile 2, n = 886 | 1.609 (1.253–2.066) P<0.001 | 1.578 (1.229–2.026) P<0.001 | 1.446 (1.123–1.863) P=0.004 |
|  | Tertile 3, n = 887 | 2.142 (1.686–2.721) P<0.001 | 2.089 (1.644–2.654) P<0.001 | 1.622 (1.256–2.094) P<0.001 |
|  | **SBPV** | 1.076 (1.059–1.093) P<0.001 | 1.073 (1.056–1.091) P<0.001 | 1.069 (1.050–1.087) P<0.001 |
|  | SBPV tertiles | P for trend < 0.001 | P for trend < 0.001 | P for trend < 0.001 |
|  | Tertile 1, n = 886 | Ref | Ref | Ref |
|  | Tertile 2, n = 886 | 1.075 (0.836–1.381) P=0.575 | 0.990 (0.768–1.277) P=0.941 | 0.876 (0.676–1.134) P=0.313 |
|  | Tertile 3, n = 887 | 1.987 (1.587–2.488) P<0.001 | 1.787 (1.420–2.250) P<0.001 | 1.681 (1.333–2.119) P<0.001 |

Adjustment for confounders:

Model 1 was unadjusted;

Model 2 was adjusted for sex and age;

Model 3 was likewise adjusted for age, sex, smoking history, alcohol consumption, heart rate, ischemic heart disease, diabetes, heart failure, hemoglobin, platelets, white blood cells, total cholesterol, HDL-C, LDL-C, triglycerides, serum creatinine, glucose, as well as the use of antiplatelet agents and statins.
