# Supplementary material for: Synergistic mortality risk of glycemic and blood pressure variability in critical stroke: A retrospective cohort study from the MIMIC-IV database
Source: Medicine (Baltimore). 2026 Jun 26;105(26):e49291. doi: 10.1097/MD.0000000000049291 (PMC13313635; doi:10.1097/MD.0000000000049291)
Supplement: Supplementary file 9 [file medi-105-e49291-s009.docx]

**Supplement Table 4 .Cox regression and trend test for ischemic stroke**

|  |  | **Model 1** | **Model 2** | **Model 3** |
| --- | --- | --- | --- | --- |
| **28-day mortality** | GV | 1.011 (1.006–1.016) P<0.001 | 1.011 (1.006–1.016) P<0.001 | 1.003 (0.997–1.009) P=0.320 |
|  | GV tertiles | P for trend < 0.001 | P for trend < 0.001 | P for trend = 0.015 |
|  | Tertile 1, n = 1202 | Ref | Ref | Ref |
|  | Tertile 2, n = 1202 | 1.457 (1.167–1.819) P=0.001 | \| 1.417(1.135–1.769)  P=0.002 \| 1.293 \| 2.262 \| 0 \| \| --- \| --- \| --- \| --- \| \| 1.77 \| 3.022 \| 0 \| | 1.216 (0.971–1.523) P=0.089 |
|  | Tertile 3, n = 1202 | 1.768 (1.427–2.191) P<0.001 | 1.713 (1.382–2.122) P<0.001 | 1.354 (1.077–1.703) P=0.010 |
|  | SBPV | 1.005 (1.002–1.009) P=0.001 | 1.005 (1.001–1.008) P=0.008 | 1.005 (1.002–1.009) P=0.004 |
|  | SBPV tertiles | P for trend < 0.001 | P for trend < 0.001 | P for trend < 0.001 |
|  | Tertile 1, n = 1202 | Ref | Ref | Ref |
|  | Tertile 2, n = 1202 | 1.131 (0.899–1.423) P=0.292 | 1.044 (0.829–1.316) P=0.712 | 1.006 (0.798–1.268) P=0.961 |
|  | Tertile 3, n = 1202 | 1.972 (1.603–2.427) P<0.001 | 1.807 (1.465–2.228) P<0.001 | 1.666 (1.345–2.064) P<0.001 |
| **365-day mortality** | GV | 1.011 (1.007–1.015) P<0.001 | 1.011 (1.006–1.015) P<0.001 | 1.002 (0.997–1.007) P=0.445 |
|  | GV tertiles | P for trend < 0.001 | P for trend < 0.001 | P for trend = 0.005 |
|  | Tertile 1, n = 1202 | Ref | Ref | Ref |
|  | Tertile 2, n = 1202 | 1.491 (1.221–1.821) P<0.001 | 1.459 (1.195–1.782) P<0.001 | 1.211 (0.988–1.484) P=0.065 |
|  | Tertile 3, n = 1202 | 1.829 (1.508–2.218) P<0.001 | 1.784 (1.471–2.165) P<0.001 | 1.357 (1.104–1.668) P=0.004 |
|  | SBPV | 1.006 (1.004–1.009) P<0.001 | 1.006 (1.003–1.008) P<0.001 | 1.006 (1.004–1.009) P<0.001 |
|  | SBPV tertiles | P for trend < 0.001 | P for trend < 0.001 | P for trend < 0.001 |
|  | Tertile 1, n = 1202 | Ref | Ref | Ref |
|  | Tertile 2, n = 1202 | 1.077 (0.877–1.322) P=0.478 | 1.014 (0.825–1.247) P=0.892 | 0.963 (0.783–1.185) P=0.724 |
|  | Tertile 3, n = 1202 | 1.921 (1.598–2.310) P<0.001 | 1.800 (1.494–2.170) P<0.001 | 1.603 (1.325–1.940) P<0.001 |

Adjustment for confounders:

Model 1 was unadjusted;

Model 2 was adjusted for sex and age;

Model 3 was likewise adjusted for age, sex, hemoglobin, white blood cells, HDL-C, LDL-C, total cholesterol, serum creatinine, heart rate, history of cerebrovascular disease, heart failure, ischemic heart disease, statin use, antiplatelet use, glucose, and systolic blood pressure.
