# Supplementary material for: Synergistic mortality risk of glycemic and blood pressure variability in critical stroke: A retrospective cohort study from the MIMIC-IV database
Source: Medicine (Baltimore). 2026 Jun 26;105(26):e49291. doi: 10.1097/MD.0000000000049291 (PMC13313635; doi:10.1097/MD.0000000000049291)
Supplement: Supplementary file 10 [file medi-105-e49291-s010.docx]

**Supplement Table 5. Cox regression and trend test for hemorrhagic stroke**

|  |  | **Model 1** | **Model 2** | **Model 3** |
| --- | --- | --- | --- | --- |
| **28-day mortality** | GV | 1.011 (1.007–1.015) P<0.001 | 1.010 (1.006–1.015) P<0.001 | 1.002 (0.995–1.009) P=0.549 |
|  | GV tertiles | P for trend < 0.001 | P for trend < 0.001 | P for trend < 0.001 |
|  | Tertile 1, n = 886 | Ref | Ref | Ref |
|  | Tertile 2, n = 886 | 0.978 (0.758–1.261) P=0.862 | \| 0.997(0.773–1.286)  P=0.981 \| 1.293 \| 2.262 \| 0 \| \| --- \| --- \| --- \| --- \| \| 1.77 \| 3.022 \| 0 \| | 1.008 (0.780–1.304) P=0.949 |
|  | Tertile 3, n = 887 | 1.884 (1.508–2.355) P<0.001 | 1.871 (1.497–2.338) P<0.001 | 1.515 (1.183–1.942) P=0.001 |
|  | SBPV | 1.005 (1.002–1.008) P=0.001 | 1.006 (1.003–1.010) P<0.001 | 1.008 (1.004–1.011) P<0.001 |
|  | SBPV tertiles | P for trend < 0.001 | P for trend < 0.001 | P for trend < 0.001 |
|  | Tertile 1, n = 886 | Ref | Ref | Ref |
|  | Tertile 2, n = 886 | 1.140 (0.870–1.493) P=0.342 | 1.045 (0.797–1.372) P=0.748 | 1.022 (0.778–1.342) P=0.878 |
|  | Tertile 3, n = 887 | 2.661 (2.106–3.361) P<0.001 | 2.379 (1.877–3.015) P<0.001 | 2.387 (1.877–3.035) P<0.001 |
| **365-day mortality** | GV | 1.012 (1.008–1.015) P<0.001 | 1.011 (1.007–1.015) P<0.001 | 1.005 (0.999–1.010) P=0.101 |
|  | GV tertiles | P for trend < 0.001 | P for trend < 0.001 | P for trend < 0.001 |
|  | Tertile 1, n = 886 | Ref | Ref | Ref |
|  | Tertile 2, n = 886 | 1.126 (0.896–1.416) P=0.308 | 1.145 (0.910–1.440) P=0.247 | 1.122 (0.890–1.414) P=0.331 |
|  | Tertile 3, n = 887 | 2.000 (1.627–2.459) P<0.001 | 1.986 (1.616–2.442) P<0.001 | 1.562 (1.243–1.962) P<0.001 |
|  | SBPV | 1.005 (1.002–1.008) P=0.001 | 1.006 (1.003–1.009) P<0.001 | 1.006 (1.003–1.009) P<0.001 |
|  | SBPV tertiles | P for trend < 0.001 | P for trend < 0.001 | P for trend < 0.001 |
|  | Tertile 1, n = 886 | Ref | Ref | Ref |
|  | Tertile 2, n = 886 | 1.134 (0.894–1.439) P=0.298 | 1.060 (0.834–1.347) P=0.632 | 1.027 (0.808–1.307) P=0.826 |
|  | Tertile 3, n = 887 | 2.425 (1.967–2.988) P<0.001 | 2.220 (1.795–2.744) P<0.001 | 2.155 (1.737–2.673) P<0.001 |

Adjustment for confounders:

Model 1 was unadjusted;

Model 2 was adjusted for sex and age;

Model 3 was likewise adjusted for age, sex, smoking history, alcohol consumption, heart rate, ischemic heart disease, diabetes, heart failure, hemoglobin, platelets, white blood cells, total cholesterol, HDL-C, LDL-C, triglycerides, serum creatinine, glucose, as well as the use of antiplatelet agents and statins.
