# Supplementary material for: Synergistic mortality risk of glycemic and blood pressure variability in critical stroke: A retrospective cohort study from the MIMIC-IV database
Source: Medicine (Baltimore). 2026 Jun 26;105(26):e49291. doi: 10.1097/MD.0000000000049291 (PMC13313635; doi:10.1097/MD.0000000000049291)
Supplement: Supplementary file 11 [file medi-105-e49291-s011.docx]

**Supplement Table 6. Cox regression and trend test for ischemic stroke**

|  |  | **Model 1** | **Model 2** | **Model 3** |
| --- | --- | --- | --- | --- |
| **28-day mortality** | GV | 1.008 (1.004–1.011) P<0.001 | 1.007 (1.004–1.011) P<0.001 | 1.000 (0.995–1.005) P=0.946 |
|  | GV tertiles | P for trend < 0.001 | P for trend < 0.001 | P for trend = 0.015 |
|  | Tertile 1, n = 1202 | Ref | Ref | Ref |
|  | Tertile 2, n = 1202 | 1.520 (1.222–1.892) P<0.001 | \| 1.483(1.192–1.846)  P<0.001 \| 1.293 \| 2.262 \| 0 \| \| --- \| --- \| --- \| --- \| \| 1.77 \| 3.022 \| 0 \| | 1.235 (0.989–1.542) P=0.062 |
|  | Tertile 3, n = 1202 | 2.153 (1.752–2.646) P<0.001 | 2.101 (1.709–2.583) P<0.001 | 1.541 (1.232–1.928) P<0.001 |
|  | SBPV | 1.004 (1.002–1.007) P=0.001 | 1.004 (1.001–1.007) P=0.009 | 1.005 (1.002–1.008) P=0.002 |
|  | SBPV tertiles | P for trend < 0.001 | P for trend < 0.001 | P for trend < 0.001 |
|  | Tertile 1, n = 1202 | Ref | Ref | Ref |
|  | Tertile 2, n = 1202 | 1.438 (1.148–1.801) P=0.002 | 1.350 (1.076–1.692) P=0.009 | 1.283 (1.023–1.609) P=0.031 |
|  | Tertile 3, n = 1202 | 2.510 (2.042–3.085) P<0.001 | 2.330 (1.893–2.869) P<0.001 | 2.089 (1.692–2.580) P<0.001 |
| **365-day mortality** | GV | 1.009 (1.006–1.012) P<0.001 | 1.009 (1.006–1.012) P<0.001 | 1.002 (0.999–1.006) P=0.213 |
|  | GV tertiles | P for trend < 0.001 | P for trend < 0.001 | P for trend = 0.005 |
|  | Tertile 1, n = 1202 | Ref | Ref | Ref |
|  | Tertile 2, n = 1202 | 1.619 (1.326–1.978) P<0.001 | 1.591 (1.302–1.943) P<0.001 | 1.303 (1.064–1.596) P=0.011 |
|  | Tertile 3, n = 1202 | 2.457 (2.038–2.964) P<0.001 | 2.409 (1.997–2.906) P<0.001 | 1.725 (1.408–2.113) P<0.001 |
|  | SBPV | 1.005 (1.003–1.007) P<0.001 | 1.004 (1.002–1.007) P<0.001 | 1.005 (1.003–1.008) P<0.001 |
|  | SBPV tertiles | P for trend < 0.001 | P for trend < 0.001 | P for trend < 0.001 |
|  | Tertile 1, n = 1202 | Ref | Ref | Ref |
|  | Tertile 2, n = 1202 | 1.379 (1.132–1.681) P=0.001 | 1.318 (1.080–1.607) P=0.006 | 1.237 (1.014–1.510) P=0.036 |
|  | Tertile 3, n = 1202 | 2.342 (1.953–2.809) P<0.001 | 2.221 (1.849–2.667) P<0.001 | 1.928 (1.601–2.322) P<0.001 |

Adjustment for confounders:

Model 1 was unadjusted;

Model 2 was adjusted for sex and age;

Model 3 was likewise adjusted for age, sex, hemoglobin, white blood cells, HDL-C, LDL-C, total cholesterol, serum creatinine, heart rate, history of cerebrovascular disease, heart failure, ischemic heart disease, statin use, antiplatelet use, glucose, and systolic blood pressure.
