# Supplementary material for: Synergistic mortality risk of glycemic and blood pressure variability in critical stroke: A retrospective cohort study from the MIMIC-IV database
Source: Medicine (Baltimore). 2026 Jun 26;105(26):e49291. doi: 10.1097/MD.0000000000049291 (PMC13313635; doi:10.1097/MD.0000000000049291)
Supplement: Supplementary file 12 [file medi-105-e49291-s012.docx]

**Supplement Table 7. **Graded association between high-variability parameters and hemorrhagic stroke mortality.****

|  |  | | **Number of high variability parameters** | |
| --- | --- | --- | --- | --- |
|  |  | **None(N=1233)** | **One(N=1078)** | **Two(N=348)** |
| **28-day mortality** | Model 1 | Ref | 1.704 (1.348-2.154) P<0.001 | 3.279 (2.504-4.294) P<0.001 |
|  | Model 2 | Ref | 1.600 (1.264-2.025) P<0.001 | 3.065 (2.337-4.019) P<0.001 |
|  | Model 3 | Ref | 1.509 (1.183-1.924) P=0.001 | 2.572 (1.939-3.411) P<0.001 |
| **365-day mortality** | Model 1 | Ref | 1.483 (1.200-1.833) P<0.001 | 2.983 (2.338-3.806) P<0.001 |
|  | Model 2 | Ref | 1.400 (1.132-1.733) P=0.002 | 2.812 (2.201-3.593) P<0.001 |
|  | Model 3 | Ref | 1.286 (1.033-1.602) P=0.025 | 2.298 (1.779-2.969) P<0.001 |
