# Supplementary material for: Synergistic mortality risk of glycemic and blood pressure variability in critical stroke: A retrospective cohort study from the MIMIC-IV database
Source: Medicine (Baltimore). 2026 Jun 26;105(26):e49291. doi: 10.1097/MD.0000000000049291 (PMC13313635; doi:10.1097/MD.0000000000049291)
Supplement: Supplementary file 13 [file medi-105-e49291-s013.docx]

**Supplement Table 8.**Graded association between high-variability parameters and ischemic stroke mortality.****

|  |  | | Number of high variability parameters | |
| --- | --- | --- | --- | --- |
|  |  | None(N=1668) | One(N=1472) | Two(N=466) |
| 28-day mortality | Model 1 | Ref | 1.606 (1.327-1.943) P<0.001 | 2.470 (1.956-3.119) P<0.001 |
|  | Model 2 | Ref | 1.523 (1.257-1.844) P<0.001 | 2.372 (1.877-2.998) P<0.001 |
|  | Model 3 | Ref | 1.348 (1.107-1.642) P=0.003 | 2.038 (1.589-2.614) P<0.001 |
| 365-day mortality | Model 1 | Ref | 1.588 (1.338-1.884) P<0.001 | 2.532 (2.056-3.118) P<0.001 |
|  | Model 2 | Ref | 1.525 (1.284-1.811) P<0.001 | 2.460 (1.996-3.032) P<0.001 |
|  | Model 3 | Ref | 1.316 (1.103-1.572) P=0.002 | 2.016 (1.614-2.518) P<0.001 |
